# Molecular epidemiology of carbapenem resistant gram-negative bacilli from infected pediatric population in tertiary - care hospitals in Medellín, Colombia: an increasing problem

**DOI:** 10.1186/s12879-016-1805-7

**Published:** 2016-09-01

**Authors:** Johanna M. Vanegas, O. Lorena Parra, J. Natalia Jiménez

**Affiliations:** Línea de Epidemiología Molecular Bacteriana, Grupo de Microbiología Básica y Aplicada, Escuela de Microbiología, Universidad de Antioquia, Street 67, 53- 108, Block 5, office 135, Medellín, Colombia

**Keywords:** Gram-negative bacilli, Carbapenem resistance, Infections in children

## Abstract

**Background:**

Gram-negative bacilli are a cause of serious infections in the pediatric population. Carbapenem are the treatment of choice for infections caused by multidrug-resistant Gram-negative bacilli, but the emergence of carbapenem resistance has substantially reduced access to effective antimicrobial regimens. Children are a population vulnerable to bacterial infections and the emergence of resistance can worsen prognosis. The aim of this study is to describe the clinical and molecular characteristics of infections caused by carbapenem-resistant Gram-negative bacilli in pediatric patients from five tertiary-care hospitals in Medellín, Colombia.

**Methods:**

A cross-sectional study was conducted in five tertiary-care hospitals from June 2012 to June 2014. All pediatric patients infected by carbapenem-resistant Gram-negative bacilli were included. Clinical information for each patient was obtained from medical records. Molecular analyses included PCR for detection of *bla*_VIM_, *bla*_IMP_*bla*_NDM,_*bla*_OXA-48_ and *bla*_KPC_ genes and PFGE and MLST for molecular typing.

**Results:**

A total of 59 patients were enrolled, most of them less than 1 year old (40.7 % *n* = 24), with a previous history of antibiotic use (94.9 %; *n* = 56) and healthcare-associated infections - predominately urinary tract infections (31.0 %; *n* = 18). *Klebsiella pneumoniae* was the most frequent bacteria (47.4 %), followed by *Enterobacter cloacae* (40.7 %) and *Pseudomonas aeruginosa* (11.9 %). For *K. pneumoniae*, KPC was the predominant resistance mechanism (85.7 %; *n* = 24) and ST14 was the most common clone (39.3 % *n* = 11), which included strains closely related by PFGE. In contrast, *E. cloacae* and *P. aeruginosa* were prevailing non-carbapenemase-producing isolates (only KPC and VIM were detected in 1 and 3 isolates, respectively) and high genetic diversity according to PFGE and MLST was found in the majority of the cases.

**Conclusions:**

In recent years, increasing carbapenem-resistant bacilli in children has become in a matter of great concern. It is important to conduct systemic surveillance and take measures to prevent dissemination of multidrug-resistant bacteria.

## Background

Gram negative bacteria are responsible for a significant number of infections associated with health care in the pediatric population, especially *Klebsiella pneumoniae*, *Escherichia coli* and *Pseudomonas aeruginosa* [1].

The carbapenems are considered the last resort antibiotics used for infections caused by multi-resistant Gram-negative bacilli, due to their stability against beta-lactamases penicillinases and cephalosporinases, and their broad spectrum of action [2]. Additionally, carbapenems are often the only option in the treatment of severe infections due to the side effects of other antibiotics in the pediatric population [1].

In recent years, frequent use of carbapenems has led to the emergence of resistance mechanisms, mediated primarily by enzymes called carbapenemases [3, 4]. These enzymes are found in mobile genetic elements which afford their dissemination and further limit treatment options, because they often harbor mechanisms of resistance to other antibiotics such as fluoroquinolones and aminoglycosides, necessitating the use of highly toxic antibiotics such as colistin [3, 4].

The clinical impact of carbapenem resistance has become a public health problem around the world in terms of increased mortality, longer hospital stays, and higher costs [5]. The child population in this issue is of great concern as it is a naturally vulnerable population in which the risk may vary, depending on immunological maturity, the presence of comorbidities, the presence of invasive medical devices, and even the prior use of antibiotics [6, 7].

In Colombia, the rates of carbapenem-resistant Gram-negative bacilli have increased significantly in recent years. *Klebsiella pneumoniae* cabapenemase (KPC) has been frequently reported in Enterobacteriaceae and have started to be reported in *Pseudomonas aeruginosa* isolates, leading to KPC being considered endemic in the country [8, 9]. Additionally, outbreaks of NDM carbapenemase isolates, often associated with high levels of resistance to carbapenems and other β-lactams, have been reported in neonatal intensive care units [10].

The behavior of infections caused by carbapenem- resistant bacteria has been studied in adults [11, 12]. However, despite the serious situation, very little is known about the difference in the behavior of infections caused by carbapenem-resistant bacteria in the pediatric population to that of infections reported in the adult population [1, 13, 14].

The aim of this study, therefore, was to describe the clinical and molecular characteristics of infections caused by carbapenem-resistant Gram-negative bacilli in hospitalized children from five tertiary care institutions in Medellín, Colombia.

## Methods

### Study population

A cross-sectional study was conducted at five tertiary care hospitals located in Medellín, Colombia, from June 2012 to June 2014. Hospitals A and E are large university hospitals of 754 and 700 beds respectively, while hospitals B and C are medium-size tertiary care institutions (286 and 300 beds respectively), and hospital D is a 140-bed cardiology hospital. All patients under 15 years old and infected by carbapenem non-susceptible *Pseudomonas aeruginosa, Klebsiella pneumoniae*, *Enterobacter cloacae* or *Acinetobacter baumannii* were included [15]. Microbiological and molecular analyses were performed on the first bacterial isolates recovered during hospitalization.

### Clinical and epidemiological data

Both clinical and epidemiological information were obtained from the medical records of each patient. This information included sociodemographic characteristics, antimicrobial use, hospitalization and surgical history, intensive care unit (ICU) stay, type of infection, comorbidities, treatment and outcomes such as therapeutic failure, cure, and death. Infections were classified as either community or healthcare associated, according to standard epidemiological definitions established by the U.S. Centers for Disease Control and Prevention (CDC) [16].

### Bacterial identification and antibiotic susceptibility

Identification of isolates and their antibiotic susceptibilities were carried out with the Vitek 2 automated system (BioMérieux, Marcy l’Etoile, France), according to CLSI [15].

### Detection of carbapenemasas and molecular typing

The presence of carbapenemases was evaluated through PCR amplification of genes *bla*_KPC_, *bla*_VIM_, *bla*_IMP_, *bla*_NDM_ and *bla*_OXA-48_, using previously described primers and conditions [17, 18]. After PCR amplification, forward and reverse sequencing was performed. Sequences were compared with those available at GenBank (www.ncbi.nlm.nih.gov/blast/) and Lahey database (http://www.lahey.org/Studies/).

Pulse-field gel electrophoresis (PFGE) was performed using 50 U of *Spe*I, 20 U of *Xba*I and 50 U *Xba*I restriction enzime (Thermo Scientific, United States) for *P. aeruginosa, K. pneumoniae* and *E. cloacae,* respectively. DNA fragment patterns were normalized using the bacteriophage Lambda ladder PFGE marker (New England Biolabs, UK). Electrophoresis was performed on a CHEF DR III (Bio-Rad Laboratories, Hercules, CA) at 11 °C, angle 120° and voltage gradient 6 V/cm. Cluster analysis was performed using the Dice coefficient in BioNumerics software version 6.0 (Applied Maths, Sint-Martens-Latem, Belgium). Dendrograms were generated by the unweighted pair group method using average linkages (UPGMA), with 1 % tolerance and 0.5 % optimization settings. A similarity cutoff of ≥80 % was used to define genetically related strains.

Multilocus sequence typing (MLST) was performed using the methodology previously described on a subset of isolates representing the most frequent PFGE patterns in *P. aeruginosa* and *K. pneumoniae* [19, 20]. Allele numbers and sequence types (ST) were assigned using the database maintained at http://pubmlst.org/paeruginosa/ and http://bigsdb.web.pasteur.fr/klebsiella/klebsiella.html.

### Statistical analyses

Categorical variables were described using absolute and relative frequencies. Median and interquartile range or mean and standard deviation were used for continuous variables, according to data distribution. Statistical analyses were carried out using the software package SPSS® v20.0 (SPSS Inc., Chicago, USA).

## Results

### Clinical and epidemiological characteristics

Of a total about 673 pediatric patients infected by *K. pneumoniae, E. cloacae, P. aeruginosa* and *A. baumannii* during the study period, 59 (8.8 %) were infected by carbapenem-resistant isolates and were included in this report; most of them male (55.9 %; *n* = 33) and less than 1 year old (40.7 %; *n* = 24).

*Klebsiella pneumoniae* was found to be the most frequent cause of infection in the study population (47.4 %, *n* = 28), followed by *E. cloacae* and *P. aeruginosa* (40.7 %, *n* = 24 and 11.9 %, *n* = 7; respectively). No patients infected with *A. baumannii* were observed. Hospital A contributed the largest number of cases (40.7 %; *n* = 24), followed by Hospital B (27.1 %; *n* = 16) and Hospital C (18.6 %; *n* = 11). However, *P. aeruginosa* was predominant in Hospital A (66.7 %; *n* = 16), while *K. pneumoniae* was more frequent in hospital B (39.3 % *n* = 11).

Ninety-seven percent (*n* = 57) of infections were classified as health care associated according to CDC criteria after individual assessment of cases. The most common infections were urinary tract infections (45.8 %; *n* = 27), of which 15.5 % (*n* = 9) were associated to use of urinary catheters. At the time of sample collection, 42.4 % (*n* = 25) of patients were hospitalized in the intensive care unit (ICU) and 78 % (*n* = 46) had had invasive medical device procedures, such as central venous catheters (*n* = 29), internal nutrition probes (*n* = 28), urinary catheters (*n* = 20) and mechanical ventilation (*n* = 17) (Table 1).

**Table 1:** Demographic and clinical characteristics of patients infected by carbapenem resistant Gram-negative bacilli

Characteristic	Total	*Pseudomonas aeruginosa*	*Klebsiella pneumoniae*	*Enterobacter cloacae*
	No. (%)	No. (%)	No. (%)	No. (%)
Gender				
Male	33 (55.9)	16 (66.7)	13 (46.4)	4 (57.1)
Female	26 (44.1)	8 (33.3)	15 (53.6)	3 (42.9)
Age (yrs)				
< 1	24 (40.7)	8 (33.3)	12 (42.8)	4 (57.1)
1 a 4	13 (20.0)	6 (25.0)	7 (25.0)	0
5 a 8	13 (20.0)	5 (20.8)	6 (21.4)	2 (28.6)
9 a 12	6 (10.2)	3 (12.5)	2 (7.1)	1 (14.3)
> 12	3 (5.1)	2 (8.3)	1 (3.6)	0
Hospital stay (days) Me (RI)	37 (16–76)	33 (15–70)	48 (25–77)	29 (10–33)
Hospital				
A	24 (40.7)	16 (66.7)	5 (17.9)	3 (42.9)
B	16 (27.1)	2 (8.3)	11 (39.3)	3 (42.9)
C	11 (18.6)	3 (12.5)	7 (25.0)	1 (14.3)
D	7 (11.9)	3 (12.5)	4 (14.3)	0
E	1 (1.7)	0	1 (3.6)	0
History in past 6 months				
Hospitalization	44 (74.6)	15 (62.5)	25 (89.3)	4 (57.1)
Surgery	37 (62.7)	15 (62.5)	19 (67.9)	3 (42.9)
Stay in ICU	30 (50.8)	13 (54.2)	15 (53.6)	2 (28.6)
Immunosuppressive therapy	16 (27.1)	8 (33.3)	6 (21.4)	2 (28.6)
Dialysis	8 (13.56)	3 (12.5)	5 (17.9)	0
Antimicrobial use in past	56 (94.9)	23 (95.8)	27 (96.4)	6 (85.7)
Carbapenems	22 (37.3)	7 (29.2)	13 (46.4)	2 (28.6)
Piperacillin-tazobactam	22 (37.3)	3 (12.5)	15 (53.6)	4 (57.1)
Glycopeptides	18 (30.5)	7 (29.2)	10 (35.7)	1 (14.3)
1st-generation cephalosporin	17 (28.8)	6 (25.0)	8 (28.6)	3 (42.9)
Aminoglycosides	13 (22.0)	5 (20.8)	8 (28.6)	0
4th-generation cephalosporin	10 (16.9)	3 (12.5)	6 (21.4)	1 (14.3)
Penicillin	9 (15.3)	5 (20.8)	3 (10.7)	1 (14.3)
Fluoroquinolones	9 (15.3)	0	7 (25.0)	2 (28.6)
3rd-generation cephalosporin	8 (13.6)	6 (25.0)	2 (7.1)	0
TMP-SMX	7 (11.9)	2 (8.3)	5 (17.9)	0
Macrolides	2 (3.4)	2 (8.3)	0	0
Lincosamides	2 (3.4)	0	2 (7.1)	0
Oxazolidinones	2 (3.4)	0	2 (7.1)	0
2nd-generation cephalosporin	1 (1.7)	1 (4.2)	0	0
Colistin	1 (1.7)	0	1 (3.6)	0
Monobactams	1 (1.7)	0	1 (3.6)	0
Lipopeptides	1 (1.7)	1 (4.2)	0	0
Infection type				
Health care associated	57 (96.6)	23 (95.8)	28 (100)	6 (85.7)
Community associated	2 (3.4)	1 (4.2)	0	1 (14.3)
Hospitalization in ICU at time of isolate	25 (42.4)	10 (41.7)	12 (42.9)	3 (42.9)
Medical device	46 (78.0)	20 (83.3)	20 (71.4)	6 (85.7)
Central venous catheter	29 (49.2)	12 (50.0)	14 (50.0)	3 (42.9)
Enteral nutrition	28 (47.5)	12 (50.0)	12 (42.9)	4 (57.1)
Urinary catheter	20 (33.9)	6 (25.0)	12 (42.9)	2 (28.6)
Invasive mechanical ventilation	17 (28.8)	9 (37.5)	6 (21.4)	2 (28.6)
Parenteral nutrition	8 (13.6)	3 (12.5)	5 (17.9)	0
Comorbidities	56 (94.9)	22 (91.7)	28 (100.0)	6 (85.7)
Cardiovascular disease	12 (20.3)	4 (16.7)	7 (25.0)	1 (14.3)
Neurologic disease	7 (11.9)	3 (12.5)	3 (10.7)	1 (14.3)
Lung disease	6 (10.2)	3 (12.5)	1 (3.6)	2 (28.6)
Chronic renal disease	5 (8.5)	4 (16.7)	1 (3.6)	0
Transplant	5 (8.5)	2 (8.3)	2 (7.1)	1 (14.3)
Trauma	3 (5.1)	1 (4.2)	2 (7.1)	0
Burns	3 (5.1)	2 (8.3)	1 (3.6)	0
Cancer	2 (3.4)	0	2 (7.1)	0
Leukemia	2 (3.4)	1 (4.2)	1 (3.6)	0
Cystic fibrosis	1 (1.7)	1 (4.2)	0	0
Immunosuppression	1 (1.7)	1 (4.2)	0	0
Infection site				
Urinary tract infection (UTI)	18 (31)	5 (20.8)	10 (37.0)	3 (42.9)
Catheter-associated UTI	9 (15.5)	3 (12.5)	6 (22.2)	0
Ventilator-associated pneumonia	7 (12.2)	2 (8.3)	3 (11.1)	2 (28.6)
Bloodstream	6 (10.3)	1 (4.2)	4 (14.8)	1 (14.3)
Pneumonia	6 (10.3)	5 (20.8)	1 (3.7)	0
Catheter-related bloodstream	3 (5.2)	2 (8.3)	1 (3.7)	0
Skin and soft tissue	2 (3.4)	2 (8.3)	0	0
Surgical site	1 (1.7)	0	0	1 (14.3)
Intra-abdominal	1 (1.7)	0	1 (3.7)	0
Empirical therapy	51 (86.4)	19 (79.2)	25 (89.3)	7 (100.0)
Carbapenems	16 (27.1)	4 (16.7)	11 (39.3)	1 (14.3)
Piperacilin-tazobactam	16 (27.1)	7 (29.2)	6 (21.4)	3 (42.9)
Glycopeptides	13 (22)	6 (25.0)	5 (17.9)	2 (28.6)
Aminoglycosides	13 (22)	2 (8.3)	9 (32.1)	2 (28.6)
4th-generation cephalosporin	7 (11.9)	4 (16.7)	1 (3.6)	2 (28.6)
Fluoroquinolones	3 (5.1)	1 (4.2)	2 (7.1)	0
Monobactams	2 (3.4)	2 (8.3)	0	0
3rd-generation cephalosporin	2 (3.4)	2 (8.3)	0	0
1st-generation cephalosporin	1 (1.7)	0 (0)	1 (3.6)	0
Penicillin	1 (1.7)	0	0	1 (14.3)
Oxazolidinones	1 (1.7)	0	1 (3.6)	0
TMP-SMX	1 (1.7)	0	1 (3.6)	0
Colistin	1 (1.7)	0	1 (3.6)	0
Targeted therapy	53 (89.8)	20 (83.3)	27 (96.4)	6 (85.7)
Colistin	16 (27.1)	2 (8.3)	14 (50.0)	0
Aminoglycosides	16 (27.1)	6 (25.0)	9 (32.1)	1 (14.3)
Carbapenems	14 (23.7)	3 (12.5)	10 (35.7)	1 (14.3)
Fluoroquinolones	10 (16.9)	6 (25.0)	3 (10.7)	1 (14.3)
4th-generation cephalosporin	9 (15.3)	5 (20.8)	2 (7.1)	2 (28.6)
Piperacilin-tazobactam	4 (6.8)	3 (12.5)	0	1 (14.3)
Monobactams	2 (3.4)	1 (4.2)	1 (3.6)	0
3rd-generation cephalosporin	2 (3.4)	2 (8.3)	0	0
Glycopeptides	1 (1.7)	0	1 (3.6)	0
Oxazolidinones	1 (1.7)	0	1 (3.6)	0
TMP-SMX	1 (1.7)	0	1 (3.6)	0
Surgical Treatment	6 (10.2)	3 (12.5)	3 (10.7)	0
Outcome				
Cure	38 (69.1)	13 (59.1)	20 (76.9)	5 (71.4)
Death	9 (16.4)	4 (18.2)	4 (15.4)	1 (14.3)
Improvement	7 (12.7)	4 (18.2)	2 (7.7)	1 (14.3)
Voluntary discharge	1 (1.8)	1 (4.5)	0	0

The medical histories of patients revealed the presence of comorbidities and frequent use of antibiotic within the past 6 months (94.9 %, *n* = 56), mainly carbapenems and piperacillin / tazobactam (37.3 %; *n* = 22), which were also those most commonly used as empirical treatments. Likewise, a high percentage of patients with history of hospitalization (74.6 %; *n* = 44) and surgery during the previous six months (62.7 %; *n* = 37) were found, more so in the case of *K. pneumoniae* than other bacteria (Table 1).

In the targeted therapy, the use of colistin is highlighted in the patients infected with *K. pneumoniae* (50 %; *n* = 14) and the use of aminoglycosides and fluoroquinolones of those infected with *P. aeruginosa* (25 %: *n* = 6).

The main outcome in the patients studied was cure (69.1 %; *n* = 38), however, all-cause mortality resulted in 16.4 % (*n* = 9) of cases. The median of hospital stay was higher for infections caused by *K. pneumoniae* (48 days) compared to those caused by *P. aeruginosa* and *E. cloacae* (33 and 29, respectively) (Table 1).

### Antibiotic susceptibility

**Fig. 1:**
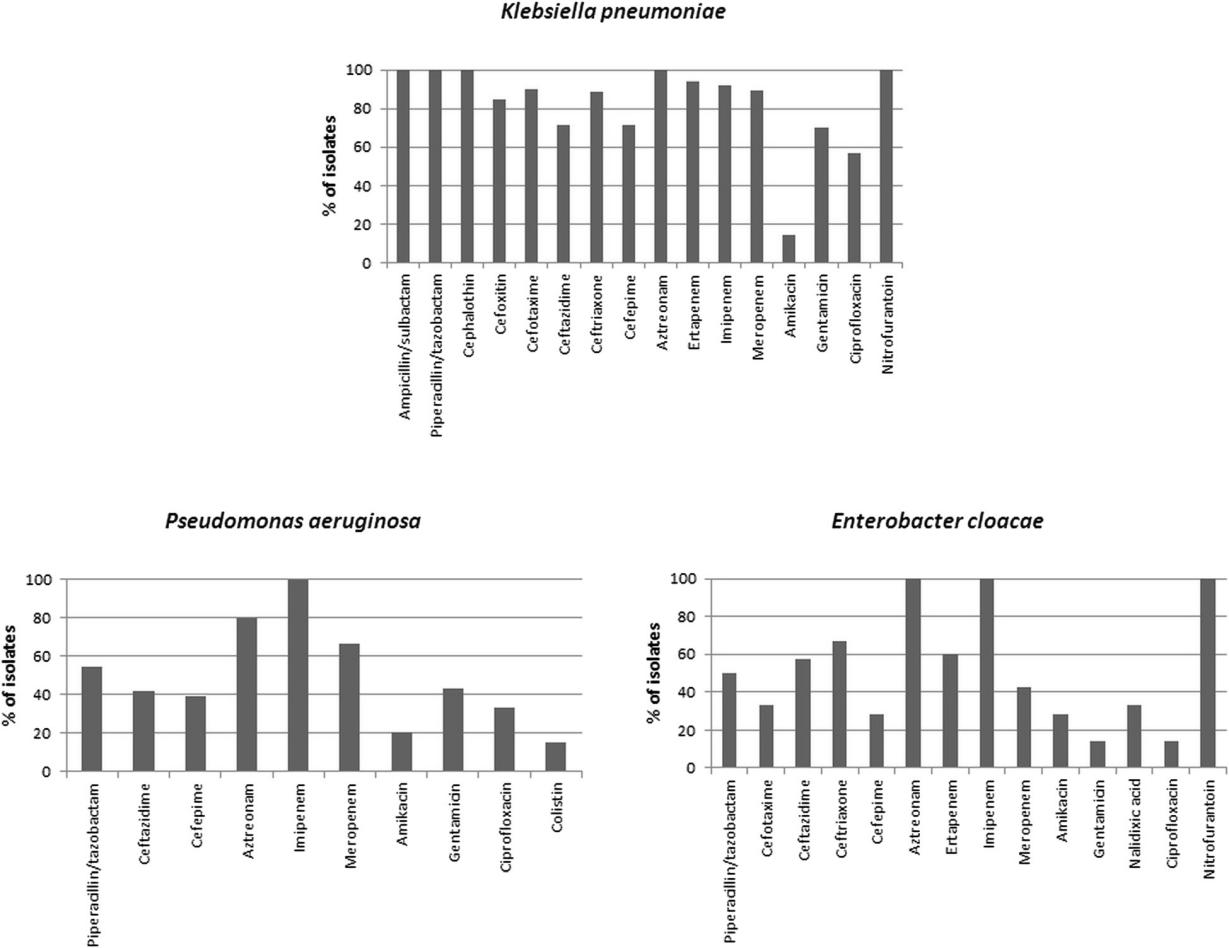
Resistance percentages in carbapenem resistant Gram-negative bacilli

In *K. pneumoniae* isolates, high frequencies of ertapenem, imipenem and meropenem resistance were observed (94.1, 92, and 89.3 %, respectively). With the exception of amikacin and ciprofloxacin, the resistance rate to all antibiotics tested was over 70 %. In contrast, in *P. aeruginosa* and *E. cloacae* isolates, imipenem resistance was observed in 100 % of cases, and for other antibiotics, including meropenem and ertapenem, resistance was under 67 % (Fig. 1).

### Carbapenemases detection and molecular typing

**Fig. 2:**
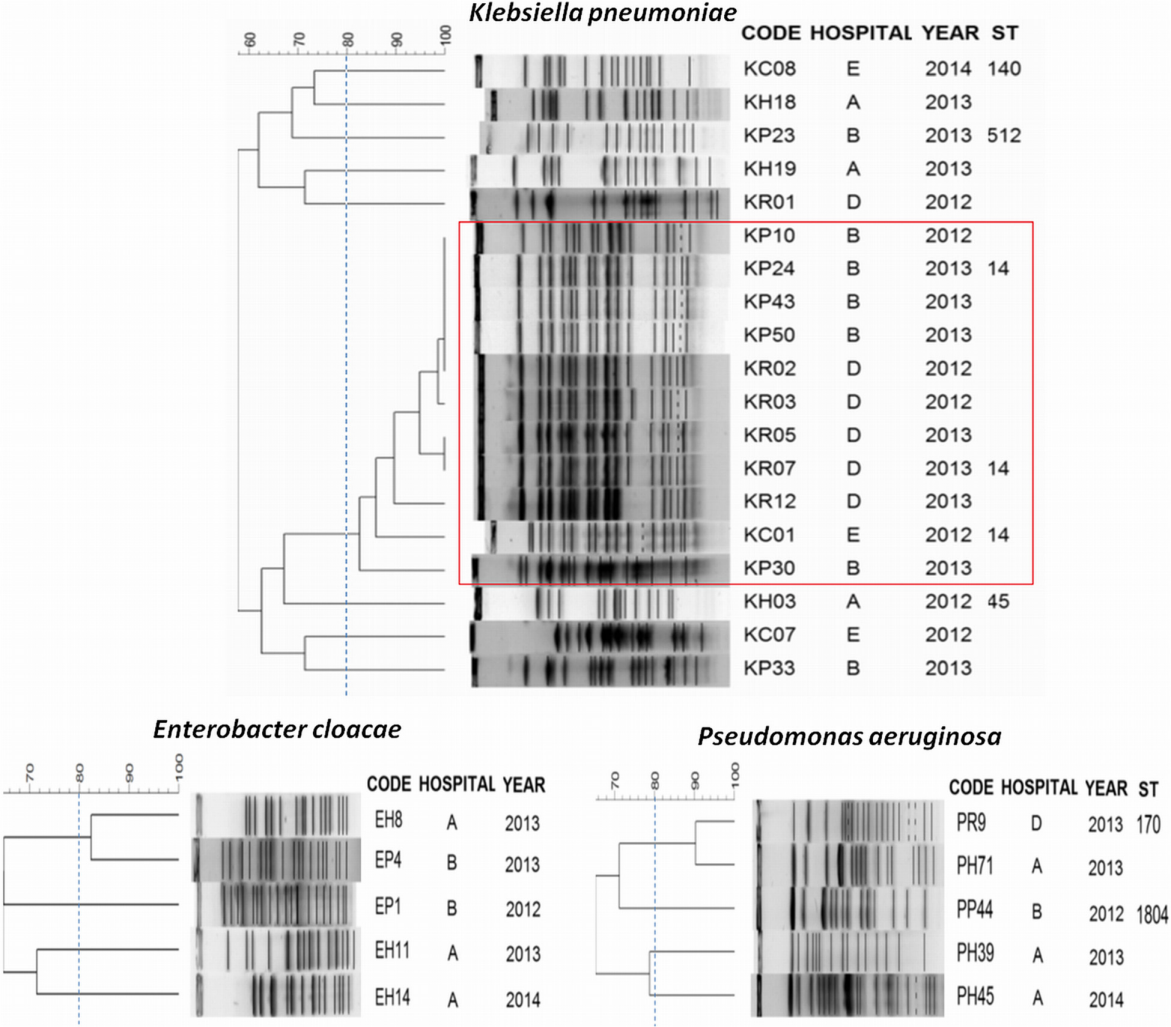
Genetic relatedness of carbapenem resistant Gram-negative bacilli

The presence of carbapenemases was observed mainly in isolates of *K. pneumoniae*, being 85.7 % (*n* = 24) positive for KPC carbapenemase, of which 21 contained KPC-2 and 3 KPC-3. For *P aeruginosa* and *E. cloacae*, most isolates were non-carbapenemase producing (87.5 %, *n* = 21 and 85.7 %; *n* = 6, respectively). Carbapenemase VIM-2 was found in three isolates of *P. aeruginosa*, and one isolate of *E. cloacae* was positive for KPC-3. Most *K. pneumoniae* isolates were closely related (Dice coefficient > 82 %) and MLST revealed isolates belonged to ST14 (39.3 % *n* = 11). The isolates of *E. cloacae* and *P. aeruginosa* were highly diverse and the ST170 and ST1804 were found in *P. aeruginosa* (Fig. 2).

## Discussion

The present study describe clinical and molecular characteristics of infections caused by carbapenem-resistant Gram-negative bacilli in children and provided an overview in order to improve our understanding of the problem become in a matter of great concern.

In this study, the majority of infections were healthcare associated, which have had a significant worldwide increase, especially in children [1, 14, 21, 22]. Different studies have reported the relationship between the hospital environment and the presence of infections caused by resistant bacteria, which has been associated with mortality rates as high as 37 % in the pediatric population [1, 14, 23]. These infections particularly affect children under one year old, a feature that was observed in this study and that can be explained by the immunological immaturity of infancy, which leads to neonatal patients being more susceptible [23–26].

In addition to the aforementioned, other factors that may facilitate infections by resistant bacteria are premature birth, low birth weight (associated with increased mortality), long hospital stays, the use of medical devices, underlying conditions, and previous contact with the hospital environment. These last three characteristics were frequently found in the patients included in this study [1, 6, 14].

Of all healthcare-associated infections, urinary tract infection has been reported as one of the most frequent both in the pediatric population and in children under one year old. It was also the most commonly found infection in this study, though the results contrast with those reported in other studies in which the most frequently occurring infections are bacteremia and pneumonia [1, 23, 25].

The proportion of catheter- associated urinary tract infections was 15 %, which shows the need to strengthen hand-hygiene, contact precautions and the replacement of invasive devices. Many of the outbreaks of infection caused by Gram-negative bacilli in neonatal and pediatric units have been the result of failures in these measures [1, 27]. Additionally, bacteria such as *K. pneumoniae* y *P. aeruginosa* have the ability to form biofilm, allowing them to adhere to different materials [1, 27].

Although bacteria-resistant infection rates are higher in ICU compared to other hospital wards, less than half of the patients in this study were hospitalized in this service, which shows the importance of epidemiological surveillance of these infections in wards other than ICU [6, 28].

Medical records show prior antibiotics use, predominantly carpabenems, in almost 95 % of patients in this study, which has been considered an independent risk factor for infections caused by resistant bacteria in both adults and children [11, 23, 29, 30]. These findings highlight the importance of establishing of antimicrobial stewardship programs as a strategy to control the spread of antibiotic-resistant bacteria in the pediatric population, and more importantly still, in neonatal and pediatric intensive care units where these medications are used the most frequently (55.8 %; 95 % CI: 50.3-61.3 %) [31].

Additionally, the use of antibiotics at an early age not only encourages the evolution of resistant bacteria, but also changes the body microbiota, which in turn has been associated with subsequent infections and even with immune disorders such as asthma, and metabolic disorders such as obesity [32]. This in turn heightens the risk of intestinal colonization by resistant bacteria, which in neonatal patients encourages the transmission of these microorganisms to their households after the hospitalization period [33].

As has been reported in other studies, this investigation found *Klebsiella pneumoniae* to be the most frequently-occurring microorganism [1, 14, 34]. For this bacteria the resistance mechanism observed in the majority of outbreaks was KPC, the enzyme encoded on mobile genetic elements such as transposon Tn4401, and which has been reported not only in the family Enterobacteriaceae, but also in non-fermetative bacilli such as *Pseudomonas aeruginosa* [8, 35, 36]. These genetic elements may also harbor resistance determinants to other antibiotics classes, as was evident in the high resistance rates of *K. pneumoniae* isolates compared with *P. aeruginosa* and *E. cloacae*, which for the most part did not contain carbapenemases, and had a higher sensitivity to aminoglycosides and meropenem [37, 38]. Within the group of aminoglycosides, amikacin sensitivity was higher than gentamicin sensitivity in isolations of *K. pneumoniae* and *P. aeruginosa*, a previously-observed characteristic in carbapenem resistant strains found in children [6].

It has been reported that the presence of carbapenemases such as KPC, further restricts the treatment that can be administered to pediatric patients because it limits therapeutic options not only within the beta-lactam groups, but also in other families of antibiotics [6, 39, 40]. The result has been antimicrobials in monotherapy or in combinations for the treatment of these infections, which can be highly toxic and can cause serious side effects in still-growing patients [39, 40].

In this study, 28/59 carbapenem-resistant isolates were positive for carbapenemases. The remained isolates negative to these enzymes could harbor other mechanisms for carbapenem resistance, including overexpression of efflux systems as MexAB-OprM for *P. aeruginosa*, overexpression of AmpC or ESBL betalactamases combined with permeability alteration (deficient expression or loss of porins) in *K. pneumoniae, E. cloacae* and *P. aeruginosa* or other carbapenemases were not evaluated [41, 42].

Another significant finding was the presence of the *K. pneumoniae* ST14 clone as the main cause of infections in the patients of the study. Previous reports have suggested the importance of this clone in the pediatric population, particularly in infants and have described it as a high-risk clone, due to its ability to spread and host resistance determinants to beta-lactams, including ESBL such as CTX -M-15 and carbapenemases such as KPC and NDM-1 [43–46].

Although the carbapenem-resistant *K. pneumoniae* ST258 clone is more widespread worldwide and has been found in outbreaks in neonatal units, it was not found in this study, agreement with previous research in Italy and Colombia, which a high frequency of non -ST258 clones were described [47, 48]. Meanwhile, the high genetic variability observed in isolates of *P. aeruginosa* and *E. cloacae* show a high antibiotic pressure that is conducive to the presence of new clones such as ST1804, reported for the first time in Colombia [8].

Finally, in this study there was not carbapenem-resistant *Acinetobacter baumannii* isolates, which is quite prevalent is other countries. Recent studies have shown a decrease in the frequency of resistant *A. baumannii* isolates in comparison with other multidrug-resistant Gram-negative bacilli causing infection in Colombia and particularly in Medellín [21, 49]. Likewise, a surveillance study conducted by our research group in five hospitals of Medellin, included only 32 carbapenem-resistant isolates during two years of study, showing the low frequency of this bacteria in our city [50].

## Conclusions

This study demonstrates an increase in the presence of Gram-negative carbapenem-resistant bacilli in the pediatric population, which has become a matter of serious concern. This mainly affects children less than 1 year old with underlying conditions, prior contact with the hospital environment, and a history of previous antibiotics use. It is important to conduct regular monitoring and establish stewardship programs of antibiotics to prevent the spread of resistant bacteria, which limit treatment options in a population particularly vulnerable to these infections.

## Abbreviations

ICU: Intensive care unit
KPC: *Klebsiella pneumoniae* cabapenemase
MLST: Multilocus sequence typing
PFGE: Pulse-field gel electrophoresis
ST: Sequence type
UPGMA: Unweighted pair group method using average linkages
UTI: Urinary tract infection

## References

[1] Berezin EN, Solórzano F, Resistance LAWGoB (2014). Gram-negative infections in pediatric and neonatal intensive care units of Latin America. J Infect Dev Ctries.

[2] Papp-Wallace KM, Endimiani A, Taracila MA, Bonomo RA (2011). Carbapenems: past, present, and future. Antimicrob Agents Chemother.

[3] Morrill HJ, Pogue JM, Kaye KS, LaPlante KL (2015). Treatment options for carbapenem-resistant enterobacteriaceae infections. Open Forum Infect Dis.

[4] Huang SR, Liu MF, Lin CF, Shi ZY (2014). Molecular surveillance and clinical outcomes of carbapenem-resistant *Escherichia coli* and Klebsiella pneumoniae infections. J Microbiol Immunol Infect.

[5] World Health Organization (2014). Antimicrobial Resistance. Global Report on Surveillance.

[6] Logan LK (2012). Carbapenem-resistant enterobacteriaceae: an emerging problem in children. Clin Infect Dis.

[7] Rojas MA, Efird MM, Lozano JM, Bose CL, Rojas MX, Rondón MA (2005). Risk factors for nosocomial infections in selected neonatal intensive care units in Colombia. South America J Perinatol.

[8] Vanegas JM, Cienfuegos AV, Ocampo AM, López L, del Corral H, Roncancio G (2014). Similar frequencies of *Pseudomonas aeruginosa* isolates producing KPC and VIM carbapenemases in diverse genetic clones at tertiary-care hospitals in Medellín, Colombia. J Clin Microbiol.

[9] Hernández C, Blanco V, Motoa G, Correa A, Maya JJ, de la Cadena E (2014). Evolución de la resistencia antimicrobiana de bacilos Gram negativos en unidades de cuidados intensivos en Colombia. Biomedica.

[10] Escobar Pérez JA, Olarte Escobar NM, Castro-Cardozo B, Valderrama Márquez IA, Garzón Aguilar MI, Martinez de la Barrera L (2013). Outbreak of NDM-1-producing *Klebsiella pneumoniae* in a neonatal unit in Colombia. Antimicrob Agents Chemother.

[11] Candevir Ulu A, Kurtaran B, Inal AS, Kömür S, Kibar F, Yapıcı Çiçekdemir H (2015). Risk factors of carbapenem-resistant *Klebsiella pneumoniae* infection: a serious threat in ICUs. Med Sci Monit.

[12] Tängdén T, Giske CG (2015). Global dissemination of extensively drug-resistant carbapenemase-producing Enterobacteriaceae: clinical perspectives on detection, treatment and infection control. J Intern Med.

[13] Vélez Echeverri C, Serna-Higuita LM, Serrano AK, Ochoa-García C, Rojas Rosas L, María Bedoya A (2014). Resistance profile for pathogens causing urinary tract infection in a pediatric population, and antibiotic treatment response at a university hospital, 2010-2011. Colomb Med (Cali).

[14] Sharland M, Saroey P, Berezin EN (2015). The global threat of antimicrobial resistance - The need for standardized surveillance tools to define burden and develop interventions. J Pediatr (Rio J).

[15] CLSI (2012). Performance Standards for Antimicrobial Susceptibility Testing: Twenty-Second Informational Supplement. CLSI document M100-S22.

[16] Horan TC, Andrus M, Dudeck MA (2008). CDC/NHSN surveillance definition of health care-associated infection and criteria for specific types of infections in the acute care setting. Am J Infect Control.

[17] Ellington MJ, Kistler J, Livermore DM, Woodford N (2007). Multiplex PCR for rapid detection of genes encoding acquired metallo-beta-lactamases. J Antimicrob Chemother.

[18] Poirel L, Walsh TR, Cuvillier V, Nordmann P (2011). Multiplex PCR for detection of acquired carbapenemase genes. Diagn Microbiol Infect Dis.

[19] Curran B, Jonas D, Grundmann H, Pitt T, Dowson CG (2004). Development of a multilocus sequence typing scheme for the opportunistic pathogen *Pseudomonas aeruginosa*. J Clin Microbiol.

[20] Diancourt L, Passet V, Verhoef J, Grimont PA, Brisse S (2005). Multilocus sequence typing of *Klebsiella pneumoniae* nosocomial isolates. J Clin Microbiol.

[21] Villalobos AP, Barrero LI, Rivera SM, Ovalle MV, Valera D (2014). Surveillance of healthcare associated infections, bacterial resistance and antibiotic consumption in high-complexity hospitals in Colombia, 2011. Biomedica.

[22] Salud OPdl. Vigilancia epidemiológica de las infecciones asociadas a la atención de la salud. Módulo III: información para gerentes y personal directivo Washington; 2012. [Available from: http://www.paho.org/hq/index.php?option=com_docman&task=doc_view&gid=19272&Itemid=. Accessed 12 Jun 2014.

[23] Arnoni MV, Berezin EN, Martino MD (2007). Risk factors for nosocomial bloodstream infection caused by multidrug resistant gram-negative bacilli in pediatrics. Braz J Infect Dis.

[24] Patel SJ, O’Toole D, Larson E (2012). A new metric of antibiotic class resistance in gram-negative bacilli isolated from hospitalized children. Infect Control Hosp Epidemiol.

[25] Abramczyk ML, Carvalho WB, Carvalho ES, Medeiros EA (2003). Nosocomial infection in a pediatric intensive care unit in a developing country. Braz J Infect Dis.

[26] Zorc JJ, Kiddoo DA, Shaw KN (2005). Diagnosis and management of pediatric urinary tract infections. Clin Microbiol Rev.

[27] Barreto S, Zambrano M, Araque M (2009). Phenotypic variations of susceptibility in *Klebsiella pneumoniae* strains of nosocomial origin and their association with biofilm formation. Invest Clin.

[28] Pérez-González LF, Ruiz-González JM, Noyola DE (2007). Nosocomial bacteremia in children: a 15-year experience at a general hospital in Mexico. Infect Control Hosp Epidemiol.

[29] Holt AF VI’t, Severin JA, Lesaffre EM, Vos MC (2014). A systematic review and meta-analyses show that carbapenem use and medical devices are the leading risk factors for carbapenem-resistant *Pseudomonas aeruginosa*. Antimicrob Agents Chemother.

[30] McLaughlin M, Advincula MR, Malczynski M, Qi C, Bolon M, Scheetz MH (2013). Correlations of antibiotic use and carbapenem resistance in enterobacteriaceae. Antimicrob Agents Chemother.

[31] Versporten A, Sharland M, Bielicki J, Drapier N, Vankerckhoven V, Goossens H (2013). The antibiotic resistance and prescribing in European Children project: a neonatal and pediatric antimicrobial web-based point prevalence survey in 73 hospitals worldwide. Pediatr Infect Dis J.

[32] Gibson MK, Crofts TS, Dantas G (2015). Antibiotics and the developing infant gut microbiota and resistome. Curr Opin Microbiol.

[33] Strenger V, Feierl G, Resch B, Zarfel G, Grisold A, Masoud-Landgraf L (2013). Fecal carriage and intrafamilial spread of extended-spectrum β-lactamase-producing enterobacteriaceae following colonization at the neonatal ICU. Pediatr Crit Care Med.

[34] Çoban B, Ülkü N, Kaplan H, Topal B, Erdoğan H, Baskın E (2014). Five-year assessment of causative agents and antibiotic resistances in urinary tract infections. Turk Pediatri Ars.

[35] Nordmann P, Dortet L, Poirel L (2012). Carbapenem resistance in Enterobacteriaceae: here is the storm!. Trends Mol Med.

[36] Cuzon G, Naas T, Villegas MV, Correa A, Quinn JP, Nordmann P (2011). Wide dissemination of *Pseudomonas aeruginosa* producing beta-lactamase *bla*_KPC-2_ gene in Colombia. Antimicrob Agents Chemother.

[37] Lee GC, Burgess DS (2012). Treatment of *Klebsiella pneumoniae* carbapenemase (KPC) infections: a review of published case series and case reports. Ann Clin Microbiol Antimicrob.

[38] Cao X, Xu X, Zhang Z, Shen H, Chen J, Zhang K (2014). Molecular characterization of clinical multidrug-resistant *Klebsiella pneumoniae* isolates. Ann Clin Microbiol Antimicrob.

[39] Pogue JM, Lee J, Marchaim D, Yee V, Zhao JJ, Chopra T (2011). Incidence of and risk factors for colistin-associated nephrotoxicity in a large academic health system. Clin Infect Dis.

[40] Díaz A, Ortiz DC, Trujillo M, Garcés C, Jaimes F, Gouzy AR. Clinical Characteristics of Carbapenem Resistant *Klebsiella pneumoniae* Infections in Ill and Colonized Children in Colombia. Pediatr Infect Dis J. 2016;35(3):237-41. doi:10.1097/INF.0000000000000987. 10.1097/INF.0000000000000987 26569194

[41] Lee JY, Ko KS (2012). OprD mutations and inactivation, expression of efflux pumps and AmpC, and metallo-β-lactamases in carbapenem-resistant Pseudomonas aeruginosa isolates from South Korea. Int J Antimicrob Agents.

[42] Chung HS, Yong D, Lee M (2016). Mechanisms of ertapenem resistance in Enterobacteriaceae isolates in a tertiary university hospital. J Investig Med.

[43] Arena F, Giani T, Becucci E, Conte V, Zanelli G, D’Andrea MM (2013). Large oligoclonal outbreak due to *Klebsiella pneumoniae* ST14 and ST26 producing the FOX-7 AmpC β-lactamase in a neonatal intensive care unit. J Clin Microbiol.

[44] Mshana SE, Hain T, Domann E, Lyamuya EF, Chakraborty T, Imirzalioglu C (2013). Predominance of *Klebsiella pneumoniae* ST14 carrying CTX-M-15 causing neonatal sepsis in Tanzania. BMC Infect Dis.

[45] Chen YT, Siu LK, Tsai YK, Lin FM, Koh TH, Chen JH. A Common Flanking Region in Promiscuous Plasmids Encoding *bla*_NDM-1_ in *Klebsiella pneumoniae* Isolated in Singapore. Microb Drug Resist. 2016;22(2):109-14. doi:10.1089/mdr.2015.0132. 10.1089/mdr.2015.0132 26308279

[46] Stillwell T, Green M, Barbadora K, Ferrelli JG, Roberts TL, Weissman SJ (2015). Outbreak of KPC-3 Producing Carbapenem-Resistant *Klebsiella pneumoniae* in a US Pediatric Hospital. J Pediatric Infect Dis Soc.

[47] Giuffrè M, Bonura C, Geraci DM, Saporito L, Catalano R, Di Noto S (2013). Successful control of an outbreak of colonization by *Klebsiella pneumoniae* carbapenemase-producing *K. pneumoniae* sequence type 258 in a neonatal intensive care unit, Italy. J Hosp Infect.

[48] Ocampo AM, Chen L, Cienfuegos AV, Roncancio G, Chavda KD, Kreiswirth BN (2015). High frequency of non-CG258 clones of carbapenem-resistant *Klebsiella pneumoniae* with distinct clinical characteristics: A two-year surveillance in five Colombian tertiary care hospitals. Antimicrob Agents Chemother.

[49] Maldonado NA, Múnera MI, López JA, Sierra P, Robledo C, Robledo J (2014). Trends in antibiotic resistance in Medellín and municipalities of the Metropolitan Area between 2007 and 2012: Results of six years of surveillance. Biomedica.

[50] Vanegas JM, Higuita LF, Vargas C, Cienfuegos AV EAR, Roncancio GE (2015). Carbapenem resistant Acinetobacter baumannii causing osteomyelitis and skin and soft tissue infections in hospitals in Medellín, Colombia. Biomedica.

